# Detection of Ventricular Fibrillation Based on Ballistocardiography by Constructing an Effective Feature Set

**DOI:** 10.3390/s21103524

**Published:** 2021-05-19

**Authors:** Rongru Wan, Yanqi Huang, Xiaomei Wu

**Affiliations:** 1 Center for Biomedical Engineering, School of Information Science and Technology, Fudan University, Shanghai 200433, China; rrwan19@fudan.edu.cn (R.W.); yqhuang@fudan.edu.cn (Y.H.); 2 Key Laboratory of Medical Imaging Computing and Computer Assisted Intervention (MICCAI) of Shanghai, Fudan University, Shanghai 200032, China; 3 Shanghai Engineering Research Center of Assistive Devices, Shanghai 200093, China

**Keywords:** ballistocardiography, ventricular fibrillation, feature extraction, machine learning, classification

## Abstract

Ventricular fibrillation (VF) is a type of fatal arrhythmia that can cause sudden death within minutes. The study of a VF detection algorithm has important clinical significance. This study aimed to develop an algorithm for the automatic detection of VF based on the acquisition of cardiac mechanical activity-related signals, namely ballistocardiography (BCG), by non-contact sensors. BCG signals, including VF, sinus rhythm, and motion artifacts, were collected through electric defibrillation experiments in pigs. Through autocorrelation and S transform, the time-frequency graph with obvious information of cardiac rhythmic activity was obtained, and a feature set of 13 elements was constructed for each 7 s segment after statistical analysis and hierarchical clustering. Then, the random forest classifier was used to classify VF and non-VF, and two paradigms of intra-patient and inter-patient were used to evaluate the performance. The results showed that the sensitivity and specificity were 0.965 and 0.958 under 10-fold cross-validation, and they were 0.947 and 0.946 under leave-one-subject-out cross-validation. In conclusion, the proposed algorithm combining feature extraction and machine learning can effectively detect VF in BCG, laying a foundation for the development of long-term self-cardiac monitoring at home and a VF real-time detection and alarm system.

## 1. Introduction

Sudden cardiac death (SCD) is a major global public health burden, accounting for 50% of cardiovascular deaths [1,2]. The onset of SCD is uncertain and can occur in all kinds of people, including healthy people and people with stable pre-existing conditions, in any place. According to statistics [3], most SCDs occur outside the hospital. Up to 80% of SCDs are caused by ventricular arrhythmias [4,5], of which ventricular fibrillation (VF) is the primary cause [6]. VF is the rapid, disordered, and asynchronous contraction of ventricular muscles, which is manifested as irregular fluctuation in time and space on an electrocardiogram (ECG). After the onset of VF, the patient’s ventricular blood output is sharply reduced, circulation is interrupted, and sudden death will occur within minutes if not timely intervened [6,7]. Electric defibrillation is the only effective method for the treatment of VF, and the likelihood of success is inversely proportional to the time interval between the onset of VF and the discharge operation. The success rate of resuscitation decreases by nearly 10% for each minute of delay [8,9,10]. Therefore, the timely and accurate detection of VF has very important practical significance.

Patients at high risk for SCD are indicated for implantable cardioverter defibrillators, which use intracardiac electrograms to predict VF and provide appropriate treatment [5,11]. However, many cases of VF occur in people without previous diagnosis records, and ECG is currently the most commonly used and effective tool for detecting cardiac status, which is widely used in various telemedicine and mobile health systems, including but not limited to ambulatory Holter recording and automatic external defibrillators (AEDs) [7,10,12]. These ECG analysis systems usually involve one or more VF detection algorithms, such as time-based algorithms including threshold crossing intervals algorithm [13], autocorrelation regression test [14], template matching [15], and heart rate variability analysis [16], frequency-based algorithms including VF-filter leakage measure [17] and frequency spectrum analysis [18], as well as wavelet transform [19], machine learning [20], neural network [21], and other advanced methods in recent years. However, more than 70% of OHCA occurred at home, and nearly 50% of cases were not witnessed [3]. Existing systems involving ECG signal analysis are difficult to perform long-term and non-intrusive monitoring of cardiac activity at home because they require electrodes, which may be inconvenient and uncomfortable for patients. Based on the problem, a variety of measurement techniques without disturbing the daily activities of the patients have been introduced to achieve the purpose of home health monitoring anytime and anywhere [22].

Research on non-invasive and unobtrusive monitoring of cardiorespiratory activity usually adopts ambient sensing methods; that is, there is no need to connect the sensor directly to the user’s skin, no special clothing is required, no additional operation or cooperation is required for signal measurement, and physiological signals can even be monitored without the user’s awareness [23,24]. In general, these monitoring methods rely on some form of mechanical coupling between the subject and the sensor. As early as the beginning of the 20th century, there have been some studies on the measurement of cardiac motion such as displacement, velocity, and acceleration [25], among which the typical methods include ballistocardiography (BCG) [25,26] and seismocardiography (SCG) [27,28]. The former is a measure of systemic recoil in response to cardiac ejection and the latter is a measure of local chest vibration caused by the heartbeat. Although they can measure mechanical responses to different aspects of cardiac activity, in practice, sensors often record a superposition of the two sources [24]. In view of the ambiguous definition of related terms, this manuscript uses the broadest term—BCG, to characterize the repetitive vibration signals generated by mechanical activity of the heart.

BCG, which changes with the movement of the body’s center of mass during cardiac contraction and ejection, is a contactless measurement that provides information about the overall performance of the circulatory system [29]. BCG has attracted a lot of interest in recent years due to the revolution in information technology. Various types of sensors are available for BCG signal acquisition, such as piezoelectric polyvinylidene fluoride (PVDF) sensor, electromechanical film-based sensor, pneumatic sensor, optical fiber sensor, and so on. These sensors can be embedded in the patient’s surrounding environment, such as bedposts, mattresses, cushions, backrests, and weighing scales [30], and they can be used in standing, sitting, and lying positions to monitor cardiac function while resting, working, or sleeping, without causing psychological stress or attentional responses. In general, the output of the BCG sensor is a composite signal of cardiac activity, respiratory activity, and body movement, which needs to be separated to measure vital signs [31]. The informative components can be used for heart rate or respiratory rate estimation [32,33], sleep position or stage recognition [34,35,36], blood pressure regulation, and other physiological or pathological detection [31].

Zink [35] and Wang [36] applied BCG signals to nocturnal monitoring of sleep apnea syndrome. Their results showed that the measured average heart rate was strongly correlated with ECG reference (Pearson correlation coefficient r > 0.95), and the detection accuracy of apnea events could also reach more than 90%. Bruser [37], Lahdenoja [38], and Wen [39] et al. applied BCG signals to automatic detection of atrial fibrillation (AF) by combining the method of feature extraction and machine learning, and they achieved a 90% (even 95%) accuracy. Taken together, although the accuracy and interpretability of BCG currently make it an inadequate replacement for ECG’s gold standard status, BCG’s real-time availability and non-contact with skin make it a distinct advantage in low-demand medical environments. There is no known study of BCG for VF detection, but Klap et al. [40] recorded 8 s BCG data of VF during cardiac and respiratory monitoring using piezoelectric sensors in a hospital. Combining with synchronously recorded ECG signals, they found that the heart’s mechanical beat followed an electrical beat (i.e., changes in BCG lagged behind ECG). Periodic mechanical beat disappeared (i.e., BCG waveform became irregular) when the pace exceeded a certain threshold (i.e., fibrillation occurred) and then resumed rhythmicity a few seconds after defibrillation shock and after normal electrical beat could be observed. Thus, the BCG signal of VF has a certain degree of differentiation from that of sinus rhythm (SR), which makes it possible to study the classification between them.

In this manuscript, BCG data of VF, SR, and motion artifact (MA) from 23 pigs were collected to construct a dataset. A feature extraction method based on short-term (7 s) BCG signal for VF detection is proposed, which can be applied to a variety of machine learning algorithms, in an attempt to provide a basis for long-term non-contact home SCD detection. The article is organized as follows: Section 2 describes our animal experimental scheme and approach to signal preprocessing, feature extraction, and classifier design. Section 3 presents our study results, including feature analysis and classification. Section 4 provides discussions of our findings and potential future work. Section 5 summarizes the whole passage.

## 2. Materials and Methods

**Figure 1:**
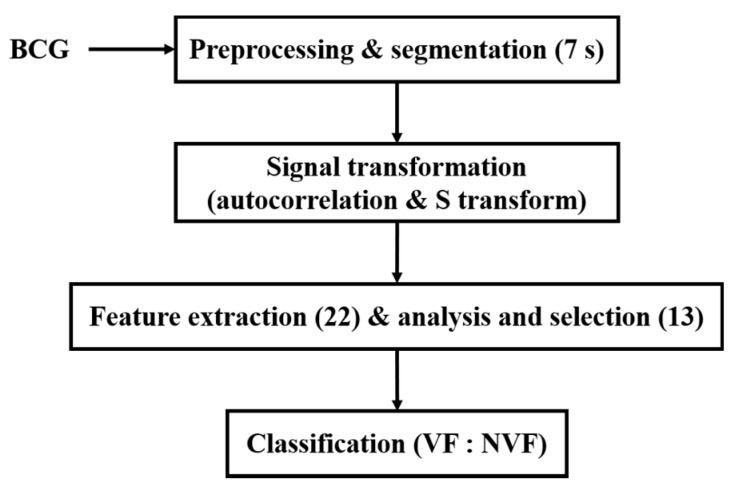
Flow diagram of the proposed method.

Due to the high risk and urgency of VF, it is quite difficult to collect BCG signals during VF from humans. BCG signals used in this study were collected from animal electric defibrillation experiment. A BCG signal is influenced by the body movement of experimental animals, the variable relative position between the animal and the sensor, as well as the individual differences, which make the BCG time-domain morphology greatly different under the same heart rhythm. Thus, it is difficult to directly extract features from the original time-domain signals to effectively distinguish VF and NVF. To this end, in this manuscript, the original BCG signal was preprocessed to remove components unrelated to the heartbeat; then, autocorrelation function (ACF) was obtained and S transform was performed. On this basis, a set of time-frequency domain features of the transformed signals were extracted, and the classification effects of two machine learning algorithms on VF and non-VF (NVF, including SR and MA) were compared. The overall flow diagram of the proposed method is shown in Figure 1.

### 2.1. Data Acquisition

In this study, BCG data of 23 experimental animals were collected in the AED electric defibrillation experiment of Jousing Medical (Suzhou) Ltd. (Suzhou, China). BCG signals including SR and VF were collected from healthy white pigs with similar physique to human. The statistical information of experimental animals is shown in Table 1. The study was approved by the Institutional Animal Care and Use Committee of Nanjing Medical University (application no. 12566, approval no. IACUC-1908001, 2019-08-02), and BCG data collection has been approved by the company. The BCG signal-gathering system, designed by Hangzhou BOBO Technology Ltd., consists of the PVDF sensor, signal conditioning circuit, and the microcontroller.

**Figure 2:**
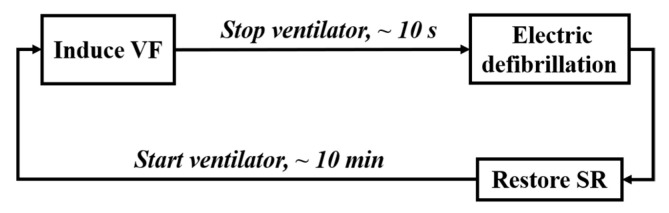
Process of the animal experiment.

The procedure of animal experiment is shown in Figure 2, including the following four steps.

(1) Animal Preparation: After intramuscular injection of basic anesthetics (zoletil), the animal’s limbs were fixed on the experimental bench with gauze strips. The anesthetic propofol was injected subcutaneously into the animal’s ear to relax the masseter muscle (about 10–15 ml), and the concentration of isoflurane was adjusted by 1% to 3% for inhalation anesthesia according to the animal condition. After skin preparation, ECG monitoring was connected, and a ventilator (frequency: 20 times/min, respiration rate: 1:2.5) was worn. The equipment integrated with BCG signal acquisition sensor was placed under the experimental mattress.

(2) Electro-induced Fibrillation: The fibrillation-induced electrode was inserted into the animal’s right ventricle through the external jugular vein. By using the VF induction device, 5 mA/50 Hz alternating current was applied to the electrode for 1 s to observe whether VF was induced. If not, the current intensity (no higher than 20 mA), the electrification time, and the position of the induced electrode would be adjusted. Stop the ventilator at the end of expiratory phase immediately after VF was induced, and set it at 20 times/min for reserve.

(3) Electric Defibrillation: Use the AED to terminate VF.

(4) Rest: Restart artificial respiration after successful defibrillation, and induce VF again after the animal maintained a stable physiological state for about 10 min.

Repeat steps (2)–(4) above. The BCG signals were recorded continuously throughout the whole process (sampling rate: 125 Hz), and the starting time of induced fibrillation and defibrillation were labeled.

### 2.2. BCG Preprocessing and Segmentation

The original BCG signals contained not only cardiac components but also respiratory components (in this experiment, not only low-frequency spontaneous breathing was involved but also pulse artifact interference caused by artificial ventilator), MA, and other noises. Although the signal conditioning circuit in the acquisition system could filter out some of the interference, further digital signal processing was needed to obtain sufficiently clean and useful signals. The large oscillation (i.e., MA) caused by the body movement of the subject will obscure the useful vibration signal generated by the cardiac activity [29]. MA epochs were manually identified and extracted based upon the non-physiological variations of the BCG’s envelope. Then, the frequency components of BCG were refined by wavelet transform to obtain useful information, and adaptive filtering was used to eliminate or suppress the regular pulse artifacts caused by ventilator.

**Figure 3:**
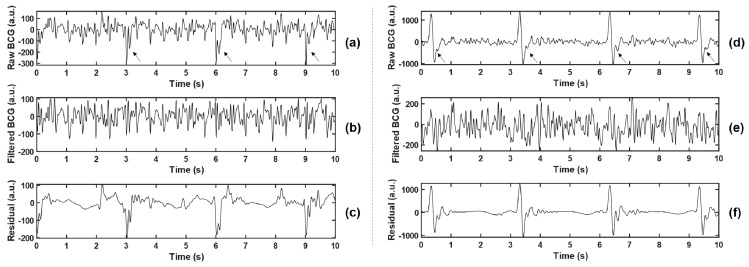
Comparison before and after adaptive filtering. (**b**,**e**) represent BCG signals after adaptive filtering in (**a**,**d**), respectively, where the arrows in (**a**,**d**) point to the pulsed artifact interference caused by the ventilator. (**c**,**f**) represent residuals between the raw BCG and the filtered BCG, namely the interference components that are filtered out.

(1) Wavelet Transform: Compared with ECG, the corresponding relationship between BCG waveform and cardiac activity is not very clear, so its regularity is not obvious. However, it usually contains a W-shaped cluster characteristic corresponding to different stages of cardiac ejection [41], and the characteristic signal is mostly distributed in the frequency band of 0.1–20 Hz. Wavelet transform can deal with nonlinear and non-stationary signals well, and it is an ideal method to analyze BCG signals. Currently, there have been many studies on the application of wavelet transform to biological signal preprocessing, including BCG-related studies [39,42]. The mother wavelet Daubechie 6 was used in our work to decompose the original BCG signal and obtain seven detail components (D1 to D7) according to the properties of each wavelet basis and its similarity with the BCG signal. Considering the frequency band information related to heartbeat in BCG and the subsequent preprocessing requirements, the BCG signal after wavelet processing was reconstructed by D3 to D7. At the same time, the D7 detail component was extracted separately, which would be used by the next filtering.

(2) Least Mean Square (LMS) Adaptive Filtering: To maintain the stability of vital signs of the animal during the experiment, it was necessary to wear a ventilator to assist ventilation (this setting does not appear in the daily monitoring of the population). The existence of ventilator made the body movement of experimental animals with each breath stronger than spontaneous breathing (but much weaker than MA), which increased the difficulty of BCG feature extraction. Observing the collected data, the interference caused by the ventilator is shown as a large pulse oscillation with a period of 3 s, as indicated by the arrows in Figure 3a,c, which cannot be effectively filtered out only by wavelet transform.

Adaptive filters have the ability to adjust their parameters automatically according to optimization algorithm, and their design requires little a priori knowledge of signal or noise characteristics [43]. To suppress the ventilator interference while leaving the useful BCG signal relatively unchanged, LMS adaptive filter was used in this article. Referring to the work of Inan et al. [44], the LMS filter algorithm is defined by Equation (1a–c):

(1a) $$y(n)=w^{T}(n-1)x(n)$$

(1b) e(n)=d(n)−y(n)

(1c) w(n)=αw(n−1)+μe(n)x(n)

where x(n), w(n), y(n), d(n), and e(n) represent the vector of buffered input samples, the vector of filter weight estimates, the filtered output, the desired response, and the estimation error (or system output) at step n, respectively. α and μ are the leakage factor and adaptation step size. The weight vector w was randomly initialized and updated iteratively by the stochastic gradient descent method to approach the optimal solution gradually. According to practice, α and μ were set to 1 and 10^−8^, and the filter length was set to 64.

The local maximum or minimum value was detected by a 3 s non-overlapping sliding rectangular window, and the large-amplitude pulse interference component caused by the ventilator was extracted, which was combined with the low-frequency component D7 obtained by wavelet decomposition, and smoothed by Savitzky–Golay (S-G) filter, so as to obtain the noise reference input of the adaptive filter. The BCG signal reconstructed by the wavelet in the previous step was taken as the desired response. Thus, the error signal was the noise-canceling BCG signal that we need. Two examples before and after the filtering are shown in Figure 3. Meanwhile, the residual signals (BCG_raw_ − BCG_filtered_, namely the interference components that were filtered out) were analyzed in the spectrum, and the main frequency peaks were tracked. The statistical results of all experimental animals (as shown in Table 2) show that the dominant frequencies of residual signals concentrated at 0.667 Hz, 1 Hz, 3 Hz, and other integer multiples of 0.333 Hz, which corresponded with the ventilation frequency of the ventilator. The filtering method effectively suppressed the non-normal waveform mutation caused by the ventilator and reduced unexpected disturbance to feature extraction.

The preprocessed BCG signals were split into 7 s segments containing 875 sample points each, corresponding with a sample rate of 125 Hz. Considering the differences in the collection of SR, MA, and VF signals during the experiment, to ensure the appropriate amount of data for each type, non-overlapping sampling was performed for SR, 3 s shift sampling was performed for MA, and 1 s shift sampling was performed for VF. BCG from 23 animals were finally obtained as follows: 7766 segments were labeled as SR, 877 segments were labeled as MA (i.e., 8643 segments were labeled as NVF), and 1356 segments were labeled as VF.

### 2.3. Signal Transformation

BCG is a combination of forces associated with blood movement in the cardiac chambers and arteries (mainly the aorta), and systolic and diastolic motion of the heart. According to the cardiac impact mechanical model, the heart causes the body to vibrate as it pumps blood, with maximum amplitude in the direction parallel to the spine. Most current measurement techniques are also largely focused on the longitudinal component from head to foot [29,30], but BCG is still essentially a 3D signal. BCG signal’s morphology varies between and within subjects, and it highly depends on the measurement device as well as the subject’s postures, which makes it difficult to give a simple quantitative explanation. Although in this study, the same supine posture was used for animal experiments, the time-domain morphology of BCG was not uniform due to objective factors such as the basic physiological differences of different experimental animals and the relative position differences between sensors and animals.

Previous studies based on BCG mostly directly used the time domain, frequency domain, complexity, and other features of the original signal for classification [37,45]. However, this method does not work on BCG signals of complex arrhythmias such as AF and VF. To solve the problem of signal diversity and highlight signal features, some studies have attempted to transform the form. For example, Wen et al. [39] converted the original BCG signals into energy signals and then extracted the features and classified them. Since the data segments used in this manuscript are only 7 s (limited by the duration of induced VF), the beats contained in each segment are far less than those in the existing BCG studies (mostly data segments with a length of 30 s or longer). To highlight the signal features, this manuscript attempted to obtain the ACF of the original BCG signal; then, it converted it from time domain to time-frequency domain and then carried out the follow-up feature extraction and classification.

(1) Wavelet Transform: The components of BCG are complex, and redundant spectral details will interfere with effective feature extraction. The heartbeat-related frequency band of BCG falls mainly between 0.7 and 15 Hz [41]. Therefore, on the basis of preprocessing, the wavelet transform was carried out again to obtain more energy-concentrated signals. The wavelet Daubechie 6 was again applied to obtain 7-layer detail components, and the reconstructed signal $BCG_{wt}$ was obtained according to Equation (2), where $d_{i}$ represents the detail component of the ith layer obtained from the decomposition of the preprocessed BCG signal, and $cc_{6}$ represents the correlation coefficient between $d_{6}$ and the preprocessed BCG signal, which is used as the weight of $d_{6}$ component to improve the deviation of BCG energy distribution caused by individual differences of subjects.

(2) $$BCG_{wt}=\left|cc_{6}\right|*d_{6}+\sum_{i=3}^{5}d_{i}$$

(2) Autocorrelation: ACF measures the cross-correlation between a signal and itself at different time. It is a useful mathematical tool to find out the repeating pattern of the signal (such as the periodic signal masked by noise) or to identify the fundamental frequency hidden in the harmonic frequency of the signal [46]. Studies have found that ACF can indeed display some unique modes of VF signals, such as aperiodicity and random amplitude [14,47]. According to the autocorrelation regression test of Chen et al. [14] based on ECG, for quasi-periodic signals, such as SR and tachycardia, ACF usually showed a regular peak value and a linear decreasing trend in most cases, while for VF, it showed scattered distribution. We suspected the same was true for BCG.

**Figure 4:**
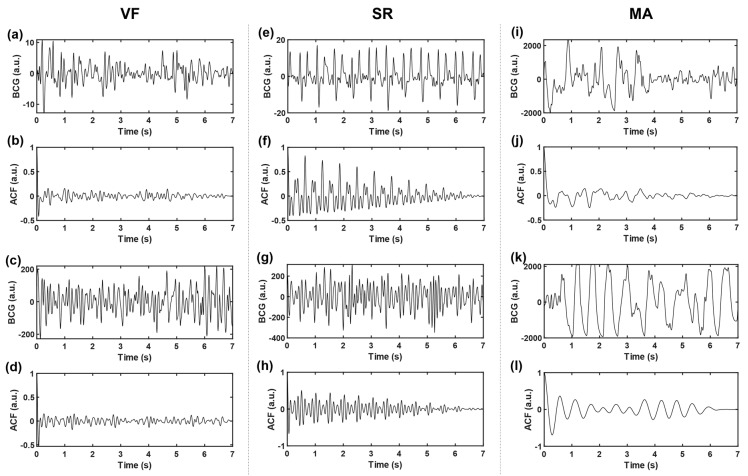
Time-domain BCG and ACF sequences. (**a**–**d**) belongs to VF, (**e**–**h**) belongs to SR, and (**i**–**l**) belongs to MA; where (**b**,**d**,**f**,**h**,**j**,**l**) represent the ACF sequences of time-domain BCG signals (**a**,**c**,**e**,**g**,**i**,**k**), respectively.

For the random process $x_{t}$, the ACF value at the sample point with lag time k is calculated according to Equation (3) [48]:

(3) $$r_{k}=\frac{1}{c_{0}N}\sum_{t=1}^{N-k}\left(x_{t}-\bar{x})(x_{t+k}-\bar{x}\right),k=0,1,\cdots ,K$$

where $c_{0}$ and $\bar{x}$ denote the sample variance and sample mean of the time series, respectively, and N represents sequence length (i.e., number of sampling points). In this work, the time delay range K=N−1. The autocorrelation sequences obtained based on three kinds of signals, VF, SR, and MA, with two cases of each kind, are shown in Figure 4. The subgraph of the first row of each kind shows two BCG time-domain waveforms from different subjects. Correspondingly, the subgraph of the second row is the ACF sequence obtained according to Equation (3), and the longitudinal axis corresponds to ACF values under different lag time (normalized, i.e., divided by the maximum value).

Compared with the original time-domain signals, ACF components are cleaner, and the “J peak” (here refers to the local maximum wave peak reflecting the heartbeat rhythm, rather than the J wave in the W-shaped cluster of BCG signals in the usual sense) is more prominent, which is conducive to the subsequent extraction of typical features. To further highlight the “J peak”, ACF was normalized to the range [1,2] and then squared.

(3) *S* Transform: *S* transform [49] is a new time-frequency representation method, which absorbs and develops the short-time Fourier transform (STFT) and continuous wavelet transform (CWT). It is based on a moving, retractable local gaussian window that provides frequency-dependent resolution and overcomes the disadvantage of fixed resolution of STFT. At the same time, the *S*-transform phase with reference to the time origin (fixed reference point) provides useful and supplementary correction information about the spectrum, which cannot be provided by the local phase information in CWT. In recent years, time-frequency analysis based on *S* transform has been used in seismic signal analysis and biomedical signal analysis [50]. Considering the diversity of BCG waveforms and the need for signal details, the ACF obtained in the previous step was tried to obtain time-frequency graph by *S* transform, which was used as the target object of feature extraction.

The one-dimensional continuous *S* transform expression of signal $x_{t}$ is shown in Equation (4a,b):

(4a) $$S_{\tau ,f}=\int_{-\infty }^{\infty }x_{t}w_{t-\tau ,f}e^{-i2\pi ft}dt$$

(4b) $$w_{t-\tau ,f}=\frac{\left|f\right|}{\sqrt{2\pi }}e^{\frac{-{\left(t-\tau \right)}^{2}f^{2}}{2}}$$

where $w_{t-\tau ,f}$ represents the Gaussian window function, which is a function of time and frequency; τ represents shift factor, which is used to control the position of the Gaussian window on the time axis t; f represents frequency; and i is an imaginary unit.

Combined with the spectrum distribution of useful components of the heartbeat in BCG signals, *S*-transform calculation was only carried out in the frequency range of 1–20 Hz. In addition, due to the large bias of the time-frequency calculation results obtained at the beginning and the end of the signal, only the middle period of the 7 s data segment was selected for subsequent analysis.

To determine the middle period and prepare for subsequent feature extraction (mainly applied in feature #1–4 in Section 2.4), the length of the heartbeat cycle, hbl (unit: sample point), was roughly estimated for each segment, according to the following steps. ① Set a variable window length to 0.35–1.2 seconds (i.e., 44–150 sample points), based on the heart rate range of 50–170 bpm. ② Under a certain window length, the beats were intercepted from the time-domain BCG signal without overlapping. A series of correlation coefficients of adjacent beats was obtained, and the mean value was calculated. ③ Repeat step 2 to get a series of mean values corresponding to different window lengths. ④ The window length corresponding to the maximum value was taken as the beat length of this segment.

For each 7 s data segment, the useful middle period was within the range of $\left[\frac{hbl/2+1}{125},\;\frac{N-hbl}{125}\right]$ seconds, which varied with the heartbeat condition in different signals, where N is the number of sample points in the original time-domain segment (i.e., 875).

### 2.4. Feature Extraction

**Figure 5:**
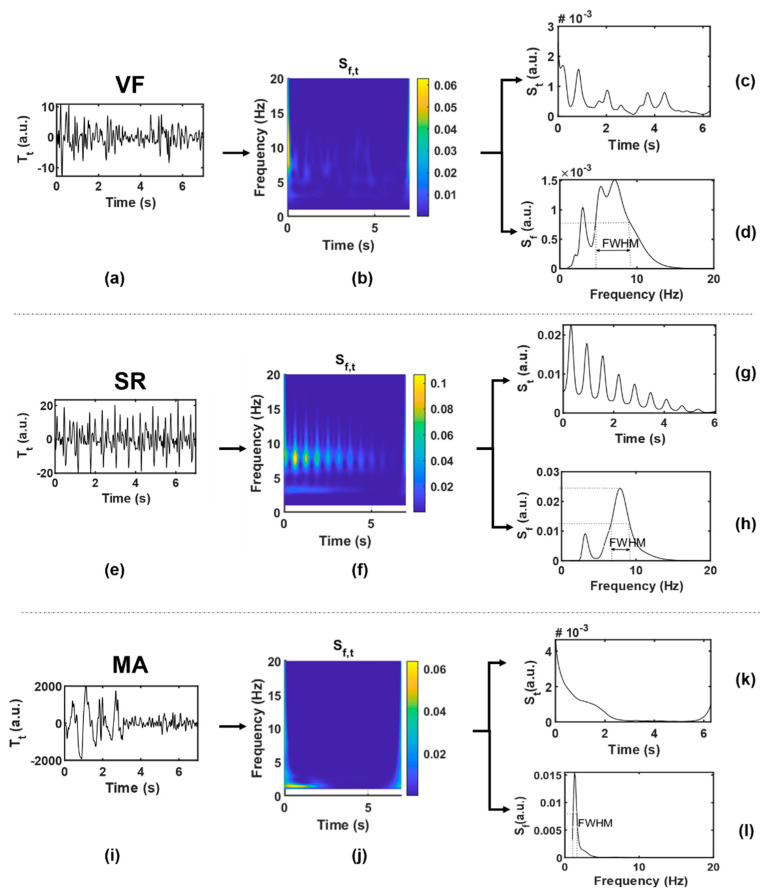
Function visualization results in the process of feature extraction. (**a**–**d**), VF; (**e**–**h**), SR; (**i**–**l**), MA, where (**a**,**e**,**i**) are original time-domain signals $T_{t}$; (**b**,**f**,**j**) are two-dimensional time-frequency signals $S_{f,t}$; (**c**,**g**,**k**) are one-dimensional signals ${\bar{S}}_{t}$ obtained by averaging along the frequency axis; (**d**,**h**,**l**) are one-dimensional signals ${\bar{S}}_{f}$ obtained by averaging along the time axis.

Twenty-two features were extracted from each data segment as potential inputs to the classifier. Table 3 lists the abbreviations and brief definitions of each feature, including 5 feature sequences (four statistics are obtained for each sequence, namely mean, variance, skewness, and kurtosis) and 2 independent feature values. For the convenience of observation, Figure 5 shows the function visualization results in the process of feature extraction, with one example for each case of VF and SR. These features will be explained in detail in terms of different functions.

(1) $S_{f,t}$: $S_{f,t}$ was given by taking the modular square of the *S*-transform result, which could be regarded as the joint distribution function of signal energy in the time-frequency domain. $S_{f,t}$ was represented by a two-dimensional matrix, with each row (j=1,…,J) corresponding to a frequency point f[j] and each column (i=1,…,I) corresponding to a time point t[i]. According to the heartbeat cycle hbl, a series of continuous slices of $S_{f,t}$ was intersected along the time axis. The correlation coefficient of each two adjacent slices was calculated to obtain the feature sequence *SC* (#1–4), as shown in Equation (5a,b). Generally speaking, BCG of SR presents synchronous rhythmic fluctuations due to rhythmic heartbeat, and the *S*-transform slices are also highly similar to each other, which are in relatively consistent time-frequency distribution, and the correlation coefficient is closer to 1 than that of VF.

(5a) $$SC[k]=corr\left(S_{f,t}\left[1:J,l_{k}\right],\;S_{f,t}\left[1:J,l_{k}+hbl\right]\right)$$

(5b) $$l_{k}=(\left(k-1\right)*hbl+1):(k*hbl),k=1,\cdots ,\left\lfloor \frac{I}{hbl}\right\rfloor -1$$

where corr means to calculate the correlation coefficient.

Combining the quasi-periodicity of SR with the irregularity of VF, the first-order moment in the frequency domain, namely the instantaneous frequency, was calculated according to Equation (6), and the feature *IF* (#5–8) was obtained. The *IF* series values obtained from VF segments are more chaotic and more widely distributed than those from SR.

(6) $$IF[i]=\frac{\sum_{j=1}^{J}f[j]*S_{f,t}[j,i]}{\sum_{j=1}^{J}S_{f,t}[j,i]},i=1,\cdots ,I$$

(2) ${\bar{S}}_{t}$*:* Average $S_{f,t}$ along the frequency axis and smooth it by S-G filter to get the function ${\bar{S}}_{t}$ of time. ACF can usually be used to represent the amplitude change intensity of time series. When the amplitude is stable and changes slowly, ACF will remain at a high value; otherwise, it will be small. The ACF of periodic signal is still periodic, while the ACF of random noise decays to zero rapidly with the increase of delay due to the uncorrelation between noise and noise. Combined with the above characteristics, the concept of BCG signal of VF is similar to random signal, and the ACF obtained basically fluctuates around the zero value except around the zero point. In the SR situation, because BCG signals contain certain periodic components, the ACF shows a slow linear decreasing trend approaching zero, and thus, the amplitude distribution of ${\bar{S}}_{t}$ is relatively balanced with a higher dispersion degree. To characterize this difference, we tried to obtain feature sequence *QA* (#9–12) by quantizing ${\bar{S}}_{t}$, which approximated a large number of discrete sample values to a small number of discrete values, so as to obtain a more concise and significant distribution. First, ${\bar{S}}_{t}$ was normalized into [0, Q] to obtain the sequence ${\bar{\bar{S}}}_{t}$, and then processed by Equation (7):

(7) $$QA\left[i\right]=\frac{\left\lfloor {\bar{\bar{S}}}_{t}\left[i\right]+\frac{1}{2}\right\rfloor }{Q},i=1,\cdots ,I$$

where Q=10.

Some studies have proposed that the peak of BCG energy signal can indirectly reflect the cardiac motion [51]. To some extent, ${\bar{S}}_{t}$ has the meaning in common with energy signal, and the wave peak obtained from it can also reflect the cardiac motion information. The peak of ${\bar{S}}_{t}$ in the SR situation can be regarded as a heartbeat, while that in the VF situation is difficult to be accurately defined. By observing the wave peak of ${\bar{S}}_{t}$ in the VF situation, it can be found that disorder and irregularity are still the most significant characteristics of VF, which may reflect the disordered mechanical activity of the ventricle and other complex effects. Therefore, the statistics of peak intervals of VF and SR are considered to be different. The time coordinate corresponding to the wave peak in ${\bar{S}}_{t}$ was defined as P[n] (n represents the order of the peak in ascending order of time); then, the feature sequence *PI* (#13–16), namely the peak interval, was obtained by Equation (8):

(8) PI[n]=P[n+1]−P[n].

(3) ${\bar{S}}_{f}$*:* Average $S_{f,t}$ along the time axis to get the function ${\bar{S}}_{f}$ of frequency. ${\bar{S}}_{f}$ can be regarded as the average power spectral density, which has different distribution in VF and SR. Function ${\bar{S}}_{f}$ of VF has smaller values and more complex spectral components. Firstly, the feature sequence *SD* (#17–20) was directly obtained from ${\bar{S}}_{f}$ without transformation, as shown in Equation (9):

(9) $$SD\left[j\right]={\bar{S}}_{f}\left[j\right],j=1,\cdots ,J.$$

The dominant frequency peak was extracted from ${\bar{S}}_{f}$. The BCG signal of SR is almost a periodic signal, and the J-wave energy is high, which is prominent in a W-shaped cluster, so there is usually a dominant frequency peak (i.e., the maximum peak of the spectrum). Ideally, the shape of the dominant frequency peak should be narrow and concentrated. However, it is generally difficult to extract a clean enough dominant frequency peak from the VF signal due to the mixing of frequency components; in other words, it often has a broad shape because it is mixed with other frequency components. *FWHM* is the peak width at half of the peak height. Through the midpoint of the peak height, make a straight line parallel to the bottom of the peak. The straight line intersects both sides of the peak at two points, and the distance between the two points is the target value. In this manuscript, *FWHM* corresponds to frequency distance, and the unit is hertz.

(4) $T_{t}$*:* The original time-domain signal without signal transformation is denoted as $T_{t}$. Only one feature value *RM* (#22) was extracted by $T_{t}$, that is, the difference between the maximum and minimum values of the time-domain sequence, also known as the range. *RM* can represent the amplitude variation range of the original time-domain signal and is mainly used to distinguish MA signals. The BCG signal of MA may have the same irregular waveform as that of VF, but due to the intervention of other non-physiological motor factors, its amplitude is much larger than that of VF and SR, which can be regarded as its most significant feature.

### 2.5. Feature Analysis and Selection

The extracted features were analyzed from two aspects in this study: the relevance between a single feature and the target (classification of VF and NVF), and the correlation between different features.

Firstly, we can address the relevance of a feature to the prediction by hypothesis testing; that is, if a certain feature shows significant differences under different values of the target, it indicates that this feature is effective in predicting the target. Since the target in this work was binary classification and the features were real-valued, the relevance was evaluated by using Mann–Whitney U-test (or Wilcoxon rank sum test) and represented by *p*-values [52]. The Mann–Whitney U-test is a kind of nonparametric hypothesis test, which does not make any assumptions about the distribution of the variables, thus ensuring the robustness of the procedure. After the hypothesis testing, a multiple testing approach—the Benjamini–Yekutieli (BY) procedure—was performed for controlling the false discovery rate (FDR).

Secondly, considering the inherent correlation among multiple features, we adopted the hierarchical clustering method to cluster the initial feature set, so that the correlation between features of different clustering classes could be small. The process of feature clustering is as follows: ① normalize the observed values of each feature; ② calculate Pearson’s correlation coefficients between each two features; ③ calculate the Euclidean distance between each two features according to the correlation coefficients, and carry out hierarchical clustering by average linkage method.

Based on the analysis of the significance and redundancy of features, some features were filtered. The selected feature subset would be used for subsequent classification.

### 2.6. Classifier Design

Currently, classification algorithms of diseases based on BCG signals are relatively limited. According to previous studies [37,38,39,45], and considering the algorithm’s speed, simplicity, and overall performance, this manuscript selected two commonly used supervised learning algorithms to evaluate their performance in binary classification between VF and NVF based on BCG, by using the features defined above. The classifiers are as follows:

(1) Logistic Regression (LR): LR is a very simple classification model, which assumes that the data obey the logistic distribution and uses the maximum likelihood estimation method to estimate the parameters. It is often used in binary classification.

(2) Random Forests (RF): RF is an extended variant of bagging, which further introduces random attribute selection into the training of decision tree based on the construction of bagging integration of decision tree. RF has the advantages of simplicity, low computational overhead, and strong generalization.

The classifiers were implemented using toolboxes and functions in MATLAB R2019b (MathWorks, Inc., Natick, MA, USA).

### 2.7. Algorithm Evaluation

For a binary classification problem, instances can be labeled as positive or negative. For binary imbalanced datasets (such as this one), the minority class (VF) is considered positive, while the majority class (NVF) is considered negative by default. The confusion matrix is shown in Table 4.

To evaluate the performance of the classifiers from multiple angles, sensitivity (SEN), specificity (SPE), precision (PRE), F1 score (F1), balanced accuracy (bACC), and Matthews correlation coefficient (MCC) were used in this manuscript. According to the confusion matrix, the above measures can be defined as follows:

(10a) $$\mathrm{SEN}=\frac{\mathrm{TP}}{\mathrm{TP}+\mathrm{FN}}$$

(10b) $$\mathrm{SPE}=\frac{\mathrm{TN}}{\mathrm{TN}+\mathrm{FP}}$$

(10c) $$\mathrm{PRE}=\frac{\mathrm{TP}}{\mathrm{TP}+\mathrm{FP}}$$

(10d) $$F1=\frac{2\times \mathrm{SEN}\times \mathrm{PRE}}{\mathrm{SEN}+\mathrm{PRE}}$$

(10e) $$\mathrm{bACC}=\frac{\mathrm{SEN}+\mathrm{SPE}}{2}$$

(10f) $$\mathrm{MCC}=\frac{\mathrm{TP}\times \mathrm{TN}-\mathrm{FP}\times \mathrm{FN}}{\sqrt{\left(\mathrm{TP}+\mathrm{FP}\right)\left(\mathrm{TP}+\mathrm{FN}\right)\left(\mathrm{TN}+\mathrm{FP}\right)\left(\mathrm{TN}+\mathrm{FN}\right)}}.$$

## 3. Results

### 3.1. Feature Analysis and Selection

**Figure 6:**
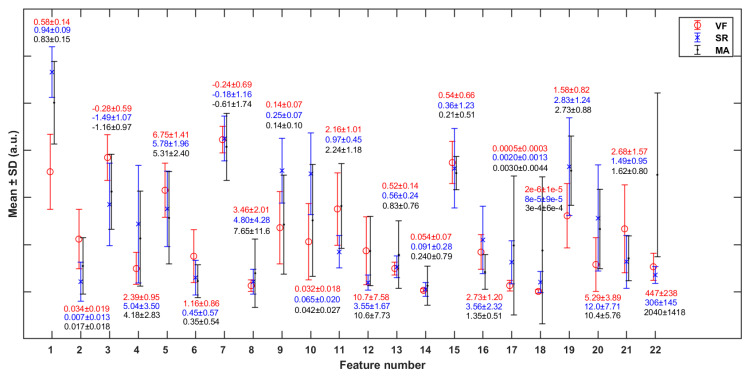
Mean and standard deviation (SD) of each of the features (in Table 2) in cases of VF, SR, and MA. The y-axis scale is normalized against the global maximum value of each specific feature (in VF, SR, and MA classes jointly).

The *p*-values representing the significant differences of each feature between any two classes are shown in Table 5. Most of the features are statistically significant in differentiating the VF class from SR, except feature 13 [*mean*(*PI*)] (*p* > 0.05). Figure 6 shows the mean and standard deviation (SD) of each feature (in Table 3) in cases of VF, SR, and MA. It can be observed that VF and SR show the greatest differences on feature 1 [*mean*(*SC*)], while VF and MA show the greatest differences on feature 22 [*RM*].

**Figure 7:**
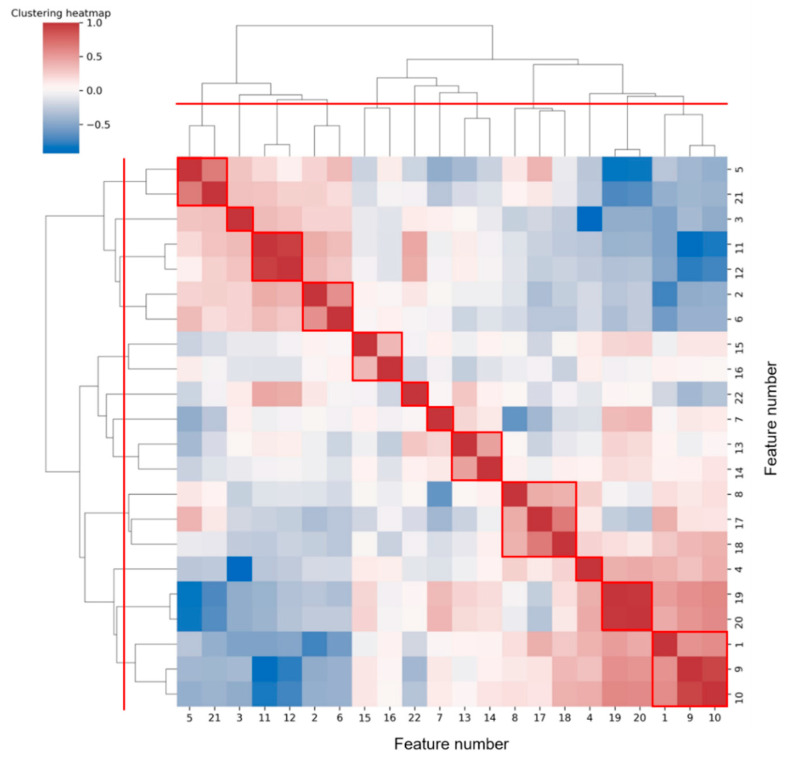
Hierarchical clustering heatmap of features. According to the red dividing line on the clustering tree, 12 classes can be aggregated, and the results are circled in red boxes.

Figure 7 shows the hierarchical clustering heatmap, in which the color represents the correlation coefficient between each two features. The color changes from blue to red, and the correlation increases.

To reduce feature redundancy, we only selected part of features from each cluster in Figure 7. The feature selection within each cluster was carried out through the corresponding statistical analysis. The hypothesis testing (as shown in Table 5) based on the median of the two sample distributions from any two classes show that feature 13 has no significant difference between VF and SR classes, while features 7, 11, 12, and 17 have no significant differences between VF and MA classes, with the FDR level of 0.05. Meanwhile, according to the mean and SD of each feature (as shown in Figure 6), in terms of features 4, 5, 7, 8, 13, 14, 15, 16, and 18, the distribution of VF class is basically included in the other two classes, especially compared with that of SR class, which makes it hard to apply these features to detect the target. Taking the above information into consideration, 13 features (1, 2, 3, 4, 7, 10, 11, 14, 16, 17, 19, 21, and 22) were finally selected from the 22 original features for subsequent work.

### 3.2. Classification Evaluation

Intra-patient and inter-patient paradigms [53] were used to evaluate the classification results. The former does not distinguish the subjects and randomly assigns the data; the latter distributes the training set and testing set according to different subjects, which is closer to the actual situation. Specifically, the intra-patient paradigm adopts the commonly used K-fold cross-validation method to avoid inaccurate assessment in extreme cases. In this manuscript, 10-fold cross-validation was used, and the data were evenly divided into subsets by systematic sampling each time. For the inter-patient paradigm, the standard LOSO cross-validation was used; i.e., training was conducted on the data of all subjects except one at a time, and then, testing was conducted on the data of the rejected subject. This process was repeated to test all subjects. 

Considering the operation time and data size, this manuscript adopted a random undersampling method to balance the difference of each class’ sample size in the training set, while the testing set was not changed. In addition, to reduce the differences caused by different sample divisions, the two evaluation paradigms were repeated 10 times, and the mean value of each evaluation index was obtained to make the final result. Table 6 and Table 7 respectively show the results of measures for the two evaluation paradigms. Overall, the classification performance of RF is better than LR.

## 4. Discussion

### 4.1. Dataset

In this study, a VF and NVF (including SR and MA) dataset was constructed. To our knowledge, this is the first time that more than one VF signal segment has been collected in BCG studies, albeit from animal experiments. The proportion of total data segments between VF and NVF is about 1:6, and there is an imbalance between samples of different classes, which is consistent with the reality. In terms of class imbalance, there are currently three main technologies: undersampling, oversampling, and cost-sensitive learning [54]. Among them, the synthetic minority oversampling technique is the representative algorithm of oversampling, that is, interpolation is carried out for the minority class to generate additional samples, but generally, it will cause high time cost. Cost-sensitive learning integrates the cost coefficient related to sample size into the classifier decision-making process, but it is often difficult to effectively infer the universal true probability based on the observation probability of the training set. The time cost of the undersampling algorithm is much lower than that of the oversampling algorithm because some samples are discarded, but some important information is also lost accordingly, which can be compensated by ensemble learning such as EasyEnsemble. In this study, considering the real-time requirement of the VF detection algorithm, a random undersampling method was adopted to solve the problem of class imbalance. The results showed that the specificity of RF could reach about 95% under the two evaluation paradigms, although some samples of the majority class (i.e., NVF) were abandoned in the training process, indicating that the features extracted in this study contained enough useful information to distinguish NVF.

Considering that the BCG data were from a limited set of investigated objects, it is necessary to discuss the overfitting problem of the model. Firstly, through statistical analysis and clustering, we selected 13 features from the original 22 features to reduce the difficulty of model learning. On the one hand, the reduction of feature dimensions from 22 to 13 improved the operation speed; on the other hand, it reduced the risk of overfitting and made the generalization ability of the model stronger. Secondly, this manuscript used LR and RF classifiers to verify the validity of the extracted features. RF, which showed better performance, introduced two random factors, namely random sampling and random selection of feature subset, in the tree construction process, thus reducing the overfitting risk of the model. Thirdly, the standard deviation of cross-validation was shown in Table 6, which was basically about 1–2%, indicating the stability of the algorithm. In addition, this study showed relatively good results in both intra-patient and inter-patient paradigms, again showing the effectiveness of the extracted features and the generalization of the algorithm.

The current NVF class in the dataset contains only SR and MA, but it does not involve other arrhythmic signals. Although ventricular tachycardia and other arrhythmias can interfere with VF detection in real situations, their effects were not considered in this study due to the lack of relevant data.

### 4.2. Methods and Results Analysis

Traditional BCG analysis algorithms were often based on the detection of W-shaped cluster features, especially J wave [32]. However, during the attack of VF, ventricular muscles are weak and contract disorderly; thus, they are unable to form a complete and regular heartbeat, and then, it is difficult to accurately locate the wave peak of BCG. In addition, it can be seen from Figure 3 and Figure 4 that even BCG signals of SR often show mixed waveforms due to the interference of other non-cardiac activities. Therefore, in the process of feature extraction in this study, BCG time-domain signals were first transformed into autocorrelation sequences from which the periodic information could be extracted more refined and directly so as to highlight the heartbeat regularity of SR and the randomness of VF. Meanwhile, the normalization step of ACF could eliminate the individual differences of amplitude among different subjects. Due to the short onset time and emergency of VF, the length of the data segment used in this study was greatly limited, which was only 7 s—much shorter than most current BCG studies (generally 30 s and longer). At this length, the feature extraction space was also limited, so this study adopted the time-frequency transformation method to expand the data dimension so as to capture useful information more comprehensively. In combination with Figure 5 and Table 3, this study extracted 21 feature values from the time-frequency graph after S transform and the one-dimensional signals obtained by averaging time-frequency graph along the time axis and frequency axis respectively, comprehensively capturing the amplitude, frequency, and morphological features of BCG signals of VF and SR. The range value extracted from the original time-domain signal was also effectively used in MA recognition. This study also tried to directly carry out S transform on the original time-domain signal without calculating ACF and then extract the above features. The results showed that the highest intra-patient accuracy could only reach about 85%, which was significantly lower than the results in Table 6. Thus, signal transformation is a necessary step of the proposed method.

By observing the results shown in Figure 6 and Figure 7, we will further explore the significance of the features extracted in this study. Firstly, we will focus on some of the features in Figure 5 that show significant distribution differences between classes. In terms of feature 1 (*mean*(*SC*)), the value for the VF class was much lower than that for NVF class. According to the definition of features in Section 2.4, feature *SC* mainly measured the regularity of heartbeat. Regarding the signals studied in this work, the heartbeat of SR had strong regularity, while that of VF had obvious irregularity. The MA signal was uncertain and usually presented as a large and slow waveform with an irregularity degree between SR and VF. The more regular the beat was, the larger the correlation value of adjacent beats was, and the more concentrated the distribution was, which led to the larger mean value (feature 1), smaller variance (feature 2), and higher kurtosis (feature 4) of the feature sequence *SC*. In terms of features #9–12 (statistical values of feature sequence *QA*), the values for VF and MA basically overlapped but differed greatly from that for SR. Feature *QA* corresponded to the amplitude distribution of the autocorrelation time series. Compared with the quasi-periodicity of SR, VF and MA signals had fewer periodicity components and were more inclined to random noise in nature. Therefore, the autocorrelation sequences obtained from these two kinds of signals would quickly decay to zero, resulting in the overall small and concentrated values of *QA* near zero, as shown in subgraphs (c) and (k) of Figure 5.

Next, we will explore the clustering results shown in Figure 7. It was common for most of the two or three features clustered into one class to be derived from different statistics of the same feature sequence. We prefer to analyze the case where features from different feature sequences were grouped together, such as clusters (5, 21), (2, 6), (8, 17, 18), and (1, 9, 10). Among them, feature 5 (*mean*(*IF*)) and feature 21 (*FWHM*) were both frequency values obtained from different angles. Both feature 2 (*variance*(*SC*)) and feature 6 (*variance*(*IF*)) assessed the regularity of heartbeat rhythm, the former from the perspective of time-frequency and the latter from the perspective of frequency domain only. Feature 8 (*kurtosis*(*IF*)) and feature 17 (*mean*(*SD*)), 18(*variance*(*SD*)) were the statistics on the distribution of different parameters (frequency and spectral density) in the frequency domain. While the extraction of feature 1 (*mean*(*SD*)) and feature 9 (*mean*(*QA*)), 10 (*variance*(*QA*)) all involved the signal periodicity. Overall, features of the same cluster were often potentially correlated.

**Figure 8:**
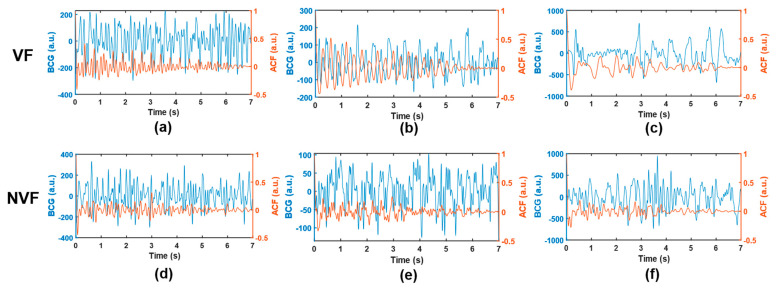
Example of misjudged signals. (**a**–**c**), VF; (**d**,**e**), SR; (**f**), MA. The BCG time-domain waveform and the corresponding ACF sequence are shown in blue and orange, respectively.

Several signals that are easy to be misjudged are selected, as shown in Figure 8. In the VF class, segments (a) and (b) were easily misjudged as SR because they involved certain rhythmicity, resulting in abnormal features such as *SC* and *FWHM*. Meanwhile, segment (c) was easily misjudged as MA due to the large amplitude and slow frequency of waveform, resulting in abnormal features such as *IF*, *SD*, and *RM*. In the NVF class, segments (d) and (e) belong to SR. As a result of noise interference, the heartbeat rhythm was not clear, and the coupling of useless components was too much, which made it difficult to extract sufficient pure and useful signals even after ACF. Segment (f) belongs to MA. Since the wave amplitude was lower than the segments of the same class and the shape was irregular, the feature *RM* did not have discrimination, and thus, it was misjudged as VF. Therefore, signals with certain regularity (but not obvious enough) or more noise components are easy to be confused between VF and SR, while signals with high amplitude (at the boundary between normal physiological vibration and external body motion) and irregularity are easy to be confused between VF and MA, which can be used as guidance for algorithm improvement.

### 4.3. Algorithm Comparison and Application

At present, no VF detection research based on BCG signals has been found, so direct comparison cannot be made. However, this manuscript will make a conceptual comparison with other similar VF detection algorithms. As mentioned in the introduction, ECG is the most widely used VF detection technology with mature algorithm and high accuracy. Tripathy et al. [55] proposed a new method combining variational mode decomposition (VMD)-based features and RF classifier for the detection of shockable ventricular arrhythmia, including ventricular tachycardia (VT) and VF, from the ECG signal, and they found the significance of the energy and entropy of each mode for detection of ventricular arrhythmia. The sensitivity and specificity values of their study were 96.5% and 97.9%, respectively, on three publicly available databases. Slama et al. [56] proposed a unique arrhythmia detection algorithm combining Fisher’s linear discriminant (FLD) and multilayer neural network (MNN) based on non-invasive ECG signals. Their method provided effective extraction of the most relevant features from non-stationary ECG signals and achieved an ideal accuracy, up to 97.38%. Mohanty et al. [57] used VMD-based features and a C4.5 classifier for the detection of ventricular arrhythmias, achieving sensitivity of 98% and specificity of 99% on two standard ECG databases with 5 s window size. They also proposed a deep neural network approach using hybrid time-frequency-based features [58] and achieved accuracy of 99% on the same databases, which can accurately characterize the VF/VT condition in the short duration signals. The 12-layer two-dimensional convolutional neural network proposed by Lai et al. [59] for detecting life-threatening ventricular arrhythmias also achieved 95% sensitivity and 99% specificity on four publicly accessible ECG databases, showing both real-time and high performance. Although the accuracy of our VF detection algorithm based on a BCG signal is slightly weaker than the above ECG-based methods, BCG can be unconsciously integrated into the living or working environment of the subject without an electrode connection, making it an advantage in home care applications, which is a strong competitive strength for BCG compared to ECG. There are also studies trying to use non-invasive wearable devices to detect VF. For example, Alonzo et al. [60] constructed a feature set by ensemble empirical mode decomposition of photoplethysmography (PPG) and combined it with the power spectral density of PPG signals. The highest accuracy and specificity obtained by K-nearest neighbor classification under 10-fold cross-validation were 84% and 92% respectively, which were significantly lower than the research results in this manuscript.

BCG does not need to put sensors in contact with the human body or wear specific devices to collect signals related to the heart and breathing, making long-term unfettered home cardiorespiratory monitoring feasible. The proposed signal transformation and feature extraction algorithm can detect VF using short-time BCG (less than 10 s) to provide a solution for unobtrusive VF detection. Currently, BCG cannot replace ECG as the gold standard for clinical diagnosis of heart because of its imperfect medical explanation, high variability among individuals or even within the same individual, and susceptibility to body movement or other external vibration interference. However, based on the results of this study, we believe that it is feasible to design a VF real-time detection and alarm system based on BCG that is suitable for the general population and is used in situations with low medical demand such as daily work or sleep. It may be possible to detect high-risk events in time and reduce the incidence of sudden death without interfering with users’ daily activities.

### 4.4. Research Limitations

This study has some limitations. First, BCG data were collected from pigs rather than from humans. Considering the diversity of BCG signals, whether the results of this study can be applied to human BCG signals needs further verification. However, the feature set and classification model constructed in this study can be used as a pre-training model for the automatic detection of VF based on human BCG signals. Second, the BCG database size is not large enough. Although a BCG-VF database containing 23 experimental animals was built in this study, which is not available in other BCG-related studies, the robustness of the model built based on the database still needs to be evaluated by more data. Third, this study only conducted a binary classification. Only SR and MA were included in the NVF class, and other ventricular arrhythmias were not considered.

## 5. Conclusions

In this manuscript, we proposed a feasible feature extraction and machine learning algorithm for VF automatic detection by BCG signals of 7 s length. The ACF of a time-domain BCG signal was obtained, and S transform was carried out to obtain a two-dimensional time-frequency graph. Based on the above transformation and statistical analysis, an effective feature set was constructed. VF was identified by machine learning algorithms, and multiple metrics were applied to evaluate algorithm performance. The results showed that the RF classifier performed well in this work, and the sensitivity and specificity were 0.965 (±0.015) and 0.958 (±0.008) under ten times 10-fold cross-validation (intra-patient paradigm), and 0.947 and 0.946 under LOSO cross-validation (inter-patient paradigm), respectively. Through the verification of human BCG signals, the proposed algorithm is expected to be applied in the BCG-based home VF real-time detection and alarm system.

## Figures and Tables

**Table 1:** Statistical information of experimental animals.

Subject Number	1–11	12–23
Age	Adult	Young
Gender (male/female)	6/5	6/6
Weight (kg)	42.1 (±5.1)	19.3 (±2.0)
Bust (cm)	76.9 (±4.2)	58.3 (±3.1)
Thoracodorsal thickness (cm)	22.3 (±2.8)	15.5 (±1.0)

**Table 2:** Statistical results of the major frequency components of residual signals for all subjects.

Subject #	Frequencies (Hz)	Subject #	Frequencies (Hz)	Subject #	Frequencies (Hz)
1	0.667, 2.67, 3	9	4.33, 4.67, 5.33	17	0.667, 1, 3
2	0.667	10	0.667, 3, 9.33	18	0.667, 1
3	0.667, 1, 3.33	11	0.667, 4, 8	19	0.667
4	0.667, 4.33, 7.67	12	0.667, 1	20	0.667, 2.67, 3
5	0.667, 2, 3.33	13	3.33	21	0.667, 3, 5.33
6	2, 2.67, 3	14	0.667, 1, 3.33	22	0.667, 1, 2.33
7	0.667, 4, 8.33	15	0.667, 1, 3	23	0.667
8	0.667, 4.33, 8	16	0.667, 2.33, 3		

**Table 3:** Brief introduction to features.

Function	#	Feature	Description
$S_{f,t}$	1–4	*SC	Slice correlation
	5–8	*IF	Instantaneous frequency
${\bar{S}}_{t}$	9–12	*QA	Quantitative amplitude
	13–16	*PI	Peak interval
${\bar{S}}_{f}$	17–20	*SD	Spectral density
	21	FWHM	Full width at half maxima of the dominant frequency peak
$T_{t}$	22	RM	Range—the difference between the maximum and the minimum

In the table, * represents the feature sequence, and the four numbers involved in each sequence correspond to mean, variance, skewness, and kurtosis in turn.

**Table 4:** Confusion matrix.

		True Condition	
		VF	NVF
Predicted condition	VF	True positive (TP)	False positive (FP)
	NVF	False negative (FN)	True negative (TN)

**Table 5:** Hypothesis testing [*p*-value (adjusted *p*-value after BY)].

#	Classes		
	VF–SR	VF–MA	SR–MA
1	<0.001	<0.001	<0.001
2	<0.001	<0.001	<0.001
3	<0.001	<0.001	<0.001
4	<0.001	<0.001	<0.001
5	<0.001	<0.001	<0.001
6	<0.001	<0.001	<0.001
7	<0.001	0.7387 (2.7266)	<0.001
8	<0.001	<0.001	<0.001 (0.0013)
9	<0.001	0.0051 (0.0241)	<0.001
10	<0.001	<0.001	<0.001
11	<0.001	0.1519 (0.6168)	<0.001
12	<0.001	0.1206 (0.5154)	<0.001
13	0.9998 (3.6899)	<0.001	<0.001
14	<0.001	0.0081 (0.0367)	<0.001
15	<0.001	<0.001	<0.001
16	<0.001	<0.001	<0.001
17	<0.001	0.2470 (0.9551)	<0.001
18	<0.001	<0.001	<0.001
19	<0.001	<0.001	0.8447 (3.1175)
20	<0.001	<0.001	0.1460 (0.5645)
21	<0.001	<0.001	<0.001
22	<0.001	<0.001	<0.001

According to the Benjamini–Yekutieli procedure, adjusted *p*-values may be greater than 1.

**Table 6:** Performance evaluation of classifiers (intra-patient, mean (standard deviation)).

	SEN	SPE	PRE	F1	bACC	MCC
LR	0.955 (0.017)	0.934 (0.008)	0.695 (0.026)	0.804 (0.017)	0.945 (0.008)	0.782 (0.019)
RF	0.965 (0.015)	0.958 (0.008)	0.783 (0.031)	0.864 (0.019)	0.961 (0.008)	0.847 (0.021)

**Table 7:** Performance evaluation of classifiers (inter-patient, mean).

	SEN	SPE	PRE	F1	bACC	MCC
LR	0.950	0.925	0.666	0.783	0.938	0.759
RF	0.947	0.946	0.735	0.828	0.947	0.806

## Data Availability

Data sharing not applicable.
